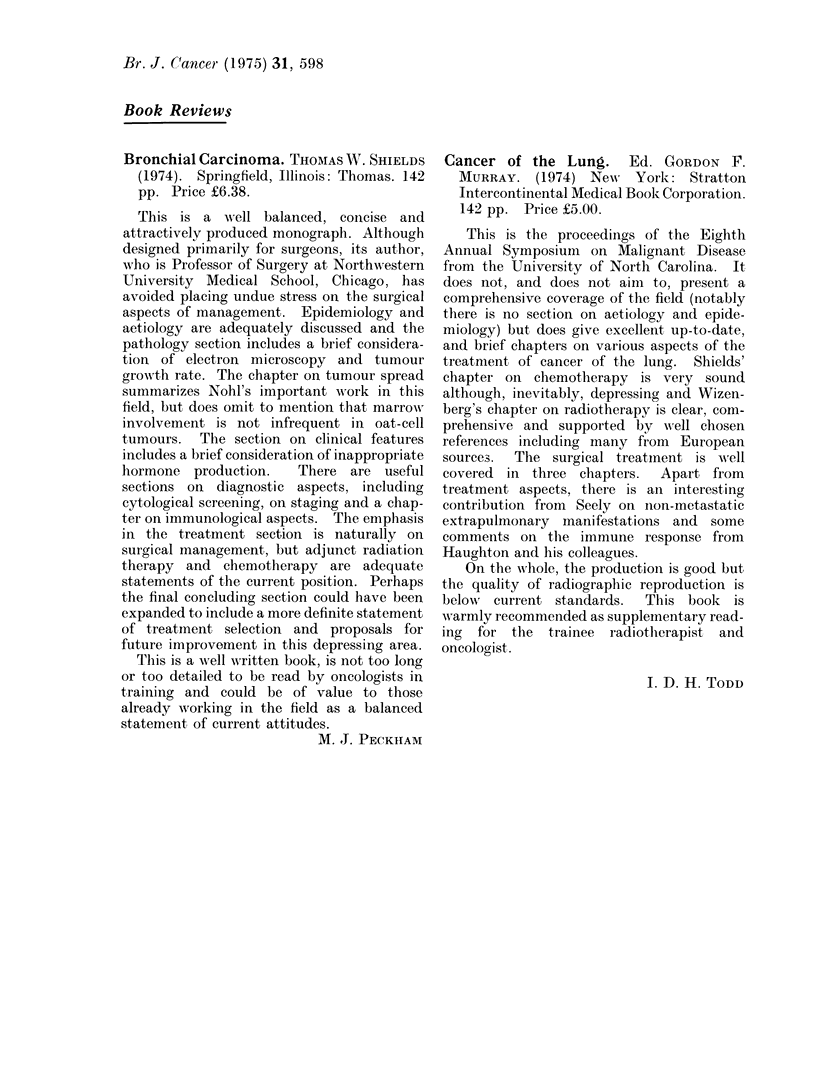# Cancer of the Lung

**Published:** 1975-05

**Authors:** I. D. H. Todd


**Cancer of the Lung.** Ed. GORDON F. MURRAY. (1974) New York: Stratton Intercontinental Medical Book Corporation. 142 pp. Price £5.00.

This is the proceedings of the Eighth Annual Symposium on Malignant Disease from the University of North Carolina. It does not, and does not aim to, present a comprehensive coverage of the field (notably there is no section on aetiology and epidemiology) but does give excellent up-to-date, and brief chapters on various aspects of the treatment of cancer of the lung. Shields' chapter on chemotherapy is very sound although, inevitably, depressing and Wizenberg's chapter on radiotherapy is clear, comprehensive and supported by well chosen references including many from European sources. The surgical treatment is well covered in three chapters. Apart from treatment aspects, there is an interesting contribution from Seely on non-metastatic extrapulmonary manifestations and some comments on the immune response from Haughton and his colleagues.

On the whole, the production is good but the quality of radiographic reproduction is below current standards. This book is warmly recommended as supplementary reading for the trainee radiotherapist and oncologist.

I. D. H. TODD